# Safety and feasibility of bilateral lung transplantation with video-assisted thoracic surgery

**DOI:** 10.1007/s00464-026-12689-6

**Published:** 2026-03-16

**Authors:** Ji Hyeon Park, Samina Park, Seon Yong Bae, Dae Hyeon Kim, Taeyoung Yun, Bubse Na, Kwon Joong Na, Hyun Joo Lee, In Kyu Park, Chang Hyun Kang, Young Tae Kim

**Affiliations:** 1 Department of Thoracic and Cardiovascular Surgery, Seoul National University Hospital, Seoul, Republic of Korea; 2 Cancer Research Institute, Seoul National University College of Medicine, Seoul, Republic of Korea; 3 Department of Thoracic and Cardiovascular Surgery, Seoul National University Hospital, Seoul National University College of Medicine, 101 Daehak-Ro, Jongno-Gu, Seoul, 03080 Republic of Korea

**Keywords:** Lung transplantation, Video-assisted thoracic surgery, Thoracotomy, Minimally invasive surgery

## Abstract

**Background:**

The clamshell incision remains the most common approach for bilateral lung transplantation because it provides excellent exposure of bilateral pleural cavities and the mediastinum. However, it is associated with significant morbidity, including sternal dehiscence and instability. Video-assisted thoracic surgery (VATS) offers a less invasive alternative. This study aimed to evaluate the feasibility, safety, and early postoperative outcomes of the VATS approach.

**Methods:**

We retrospectively analyzed 136 patients who underwent bilateral lung transplantation with extracorporeal membrane oxygenation support between August 2017 and March 2025. Patients were categorized according to surgical approach: clamshell (*n* = 105) or VATS (*n* = 31). Perioperative outcomes, complications, and pulmonary function were compared between the two modalities.

**Results:**

The VATS group had a significantly shorter operative time (319 vs. 417 min., *P* < 0.001), less blood loss (832 vs. 2,789 mL, *P* < 0.001), and required fewer transfusions. Sternal wound complications and airway interventions occurred exclusively in the clamshell group. Patients in the VATS group exhibited significantly higher pulmonary function at 1 month postoperatively than did the clamshell group (forced expiratory volume in 1 s (FEV_1_): 85.79 ± 18.00 vs. 67.25 ± 22.55, *P* < 0.001; forced vital capacity (FVC): 73.42 ± 14.14 vs. 60.00 ± 17.81, *P* < 0.001). These differences gradually attenuated but remained statistically significant at 12 months postoperatively (FEV_1_: *P* = 0.05; FVC: *P* = 0.04).

**Conclusions:**

VATS approach for bilateral lung transplantation is feasible and safe, offering lower surgical morbidity and better pulmonary function than the conventional clamshell incision. This technique may provide distinct advantages in centers with established thoracoscopic expertise.

**Graphical abstract:**

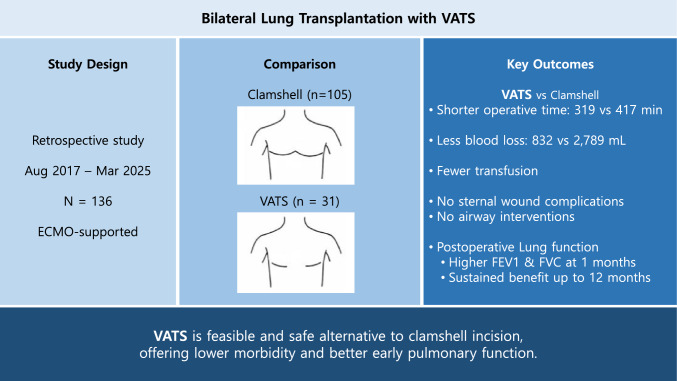

**Supplementary Information:**

The online version contains supplementary material available at 10.1007/s00464-026-12689-6.

The clamshell incision remains the most widely used surgical approach for bilateral lung transplantation because it provides excellent exposure of the bilateral pleural cavities and mediastinum, facilitating sequential implantation and intraoperative cardiopulmonary support. However, this approach is associated with significant morbidity, including sternal wound complications, dehiscence, and impaired chest wall stability, which may delay postoperative recovery. Reported rates of sternal complications range from 7 to 30%, potentially affecting early postoperative outcomes [1].

Thoracotomy approaches that preserve the sternum and internal mammary vessels have been proposed as alternatives [2–5], offering the potential to reduce wound-related morbidity and promote faster recovery of pulmonary function. However, despite these reports from several groups [2–5], such less invasive bilateral thoracotomy techniques have not gained widespread adoption due to concerns about limited mediastinal exposure, restricted angles for hilar dissection, and technical challenges in controlling the pulmonary artery and left atrium. Moreover, previous reports on minimally invasive lung transplantation have primarily described extended thoracotomies or open approaches involving relatively large incisions rather than a true video-assisted thoracic surgery (VATS) technique [2–5]. Our group previously introduced a bilateral VATS lung transplantation technique [6]. The present study builds upon that technical report by providing a comparative analysis of perioperative and early postoperative outcomes between the VATS approach and the conventional clamshell incision.

With the increasing use of extracorporeal membrane oxygenation (ECMO) for intraoperative cardiopulmonary support, the need for full mediastinal exposure has diminished. [7] This shift has encouraged the exploration of alternative surgical strategies aimed at reducing operative trauma. We developed a VATS technique employing bilateral mini-thoracotomies, endoscopic vascular clamping, and thoracoscopic instruments to perform bilateral lung transplantation while preserving sternal integrity. In this study, we describe our initial experience with this VATS approach using a mini-thoracotomy working window, which maintains sternal continuity while providing adequate access through thoracoscopic visualization and instruments. We evaluated the feasibility and safety of this approach for bilateral lung transplantation.

## Materials and methods

### Study design and ethical approval

This study was designed as a retrospective observational cohort study and was reported in accordance with the STROBE guidelines. The study protocol was reviewed and approved by the Institutional Review Board of Seoul National University Hospital as a minimal-risk retrospective study (H-2505–194-1646). The requirement for written informed consent was waived.

### Patients

We included patients who underwent bilateral lung transplantation with ECMO support between August 2017 and March 2025. Since August 2017, bilateral lung transplantation at our institution has been routinely performed under intraoperative ECMO support, either via central or peripheral cannulation, as part of our standard protocol. To ensure cohort homogeneity and minimize confounding by heterogeneous cardiopulmonary support strategies, only ECMO-supported cases were included in this analysis. ECMO support included both peripheral and central cannulation strategies, with venovenous or venoarterial configurations applied based on clinical indication. Exclusion criteria were as follows: (1) use of cardiopulmonary bypass during transplantation, (2) single-lung transplantation, and (3) multi-organ transplantation. Contraindications to the VATS approach included the following: (1) inability to apply peripheral ECMO support and (2) inability to achieve adequate lung deflation for thoracoscopic manipulation. The VATS approach was introduced in April 2024. From that time onward, VATS was adopted as the default surgical strategy for bilateral lung transplantation, except in patients meeting predefined exclusion criteria.

### Surgical approaches

**Fig. 1:**
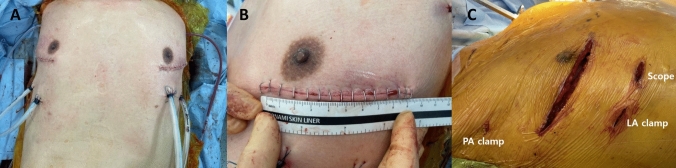
Operative setup and port configuration for VATS bilateral lung transplantation. **A** Postoperative wound appearance showing bilateral mini-thoracotomy working windows at the 4th intercostal space. The thoracoscopic port and endoscopic clamp insertion sites were used for chest tube and Jackson–Pratt drain placement. **B** Measurement of the working window length. In recent cases, the skin incision length was reduced to < 10 cm while still allowing safe donor lung insertion. **C** Intraoperative photograph showing the spatial configuration of the working window and accessory port sites. Scope and clamp insertion points are marked to indicate their positions relative to the mini-thoracotomy. *LA* left atrium, *PA* pulmonary artery, *VATS* video-assisted thoracic surgery

**Fig. 2:**
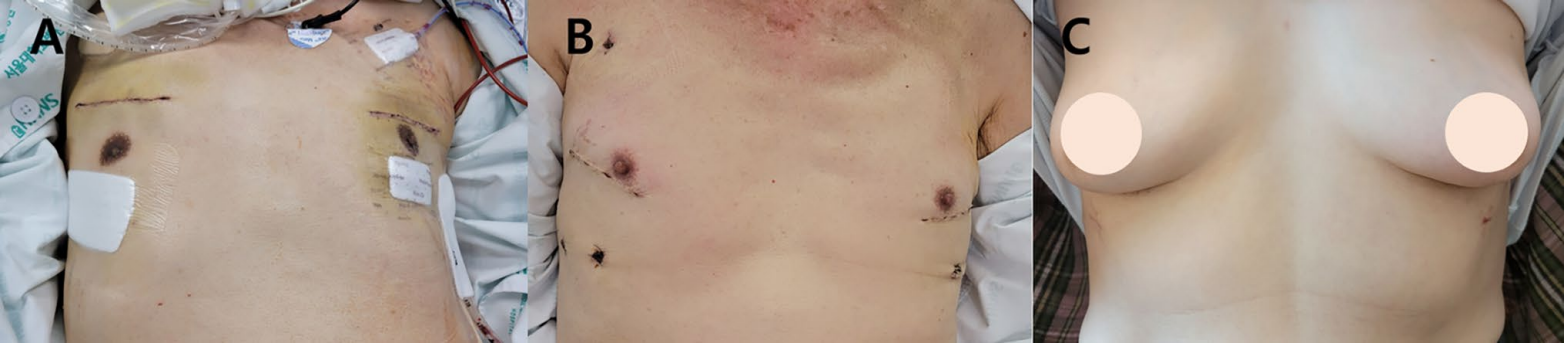
Variations in the working window incision according to patient anatomy. **A** Working incision created at the third intercostal space in patients with high hilar position. **B** Standard working incision at the fourth intercostal space. **C** In female patients, the skin incision was placed along the inframammary line, allowing the breast to naturally cover the incision while entering the thoracic cavity through the fourth intercostal space

In the VATS approach, surgery was initiated through a mini-thoracotomy serving as the working window in the fourth intercostal space (Fig. 1A, B; Supplemental Video). This incision preserved the internal thoracic artery and vein and was performed without rib cutting. The skin incision in the initial case measured approximately 13 cm; however, in recent cases, incision length of < 10 cm was sufficient for donor lung insertion using a hydrophilic vinyl bag. Depending on individual patient anatomy, including the position of the hilum or breast contour in female patients, the working window skin incision was occasionally made at the third intercostal space or placed along the inframammary line while still entering the thoracic cavity through the fourth intercostal space (Fig. 2).

A thoracoscopic camera port was placed in the fifth or sixth intercostal space and later repurposed as the chest tube site. Endoscopic vascular clamps were introduced through small additional intercostal incisions (Fig. 1C). Specifically, the pulmonary artery was clamped through the third intercostal space at the anterior axillary line, whereas the left atrium was clamped through the sixth and fifth intercostal spaces on the right and left sides, respectively, to minimize cardiac interference.

In the VATS approach, a soft tissue wound protector (NT-Port Soft Wound Retractor, Yuwon Meditech Co., Republic of Korea) was inserted at the utility incision. A rib-spreading retractor was not routinely used; it was applied only temporarily during donor lung insertion and removed immediately thereafter. The majority of hilar dissection and implantation procedures were performed under thoracoscopic visualization (indirect view). Direct visualization with surgical loupes was occasionally used as an adjunct during selected suturing steps.

Specialized thoracoscopic instruments were used to facilitate vascular control and implantation through limited access incisions. Pulmonary artery control was achieved using a double-angled VATS DeBakey-Satinsky clamp (SonTec Instrument, USA). Left atrial control was performed using thoracoscopic Lambert-Kay clamps (Wexler Surgical, USA). For vascular suturing and tissue handling, thoracoscopic graspers with ValveGate^TM^Pro DeBakey narrow straight jaws (Geister Medizintechnik GmbH, Germany), and minimally invasive needle holders (Cardio Surgical GmbH, Germany; SCANLAN, USA) were used. A double-hinged laminectomy retractor (Rebstock, Germany) was applied only temporarily during donor lung insertion and removed immediately thereafter; no sustained rib spreading was performed during the procedure. During implantation, the donor lung was introduced into the pleural cavity using a sterile insertion device (Keller Funnel, Allergan, USA), which facilitated atraumatic passage of the graft through the limited thoracotomy incision.

In the clamshell approach, a conventional transverse thoracosternotomy was performed at the fourth intercostal space, following standard practice.

Intraoperative ECMO support was used in all patients. In patients undergoing the clamshell incision, ECMO was established via central cannulation through the ascending aorta and right atrium, with femoral venous cannulation used as needed. In the VATS group, peripheral ECMO was established via femoral artery and vein cannulation using an ultrasound-guided Seldinger technique.

To minimize the risk of differential hypoxemia and optimize venous drainage during peripheral ECMO support, the venous cannula tip was positioned in the superior vena cava. Full ECMO support was maintained until completion of the first lung implantation, and the more severely diseased lung was explanted first. During implantation of the second lung, the pulmonary artery was divided as early as feasible to reduce ventilation–perfusion mismatch. When oxygenation remained insufficient after first lung implantation, conversion to a veno-arterial-venous ECMO configuration was performed.

Arterial access sites were closed using a suture-mediated closure device (Perclose ProGlide™, Abbott Cardiovascular, USA). Lower limb perfusion was continuously monitored using near-infrared spectroscopy applied to the plantar surface of the foot, and a distal perfusion catheter was inserted when prolonged postoperative VA ECMO support was anticipated.

### Operative time definitions

Warm ischemic time was defined as the interval from removal of each graft from cold storage to reperfusion of that graft. Total warm ischemic time represents the sum of the implantation warm ischemic times of both lungs.

### Statistical analysis

Continuous variables were analyzed using Student’s t-test or the Wilcoxon rank-sum test, depending on data distribution. Categorical variables were compared using the chi-square test or Fisher’s exact test, as appropriate.

Multivariable regression analyses were performed to adjust for potential baseline confounders. Covariates were selected a priori based on clinical relevance and included recipient age, sex, body mass index, pulmonary hypertension (defined as ePASP ≥ 50 mmHg), preoperative mechanical ventilation, preoperative ECMO bridging, and primary diagnosis (IPF vs non-IPF). Robust standard errors (HC3) were applied to account for heteroscedasticity. Operative time was analyzed using linear regression, whereas estimated blood loss and ICU length of stay were analyzed using log-linear regression models.

Pulmonary function was evaluated using forced expiratory volume in 1 s (FEV_1_) and forced vital capacity (FVC) measured at 1, 3, 6, 9, and 12 months after transplantation. Between-group comparisons at each time point were performed using Welch’s two-sample t-test after excluding missing values. Temporal changes in FEV_1_ and FVC were visualized using line plots, with error bars representing the standard error of the mean. To assess longitudinal changes in postoperative pulmonary function and the effect of the surgical approach, linear mixed-effects models were applied to FEV_1_ and FVC measurements. Fixed effects included surgical group (clamshell vs. VATS), time (months after surgery), and their interaction (group × time). Time was treated as a continuous variable corresponding to postoperative months (1 = baseline, 3, 6, 9, and 12 months). Models were fitted using restricted maximum likelihood estimation, and degrees of freedom were calculated using Satterthwaite’s approximation.

All statistical analyses were performed using IBM SPSS (IBM Corp., Armonk, NY) and R (version 4.4.1; R Foundation for Statistical Computing, Vienna, Austria). A *P*-value < 0.05 was considered statistically significant. Statistical analyses were performed by the authors, with the first author (J.H.P.) responsible for data processing and analysis.

## Results

### Patient characteristics

Baseline patient characteristics are summarized in Table 1. A total of 136 patients underwent bilateral lung transplantation with ECMO support during the study period. Of these, 105 patients underwent transplantation via the clamshell incision and 31 via the VATS approach. The median age was similar between the clamshell and VATS groups (60.0 years, interquartile range [IQR] 52.0–65.0 vs. 62.0 years, IQR 51.0–68.0; *P* = 0.212). The proportion of male patients did not differ significantly between groups (41.0% vs. 38.7%; *P* = 0.823). Mean body mass index was also comparable between groups (20.67 ± 4.05 vs. 20.79 ± 3.46 kg/m^2^; *P* = 0.187). The prevalence of comorbidities, including hypertension, diabetes mellitus, coronary artery disease, and cerebrovascular accidents, was similar in both groups. Idiopathic pulmonary fibrosis (IPF) was the most common indication for lung transplantation in both groups, accounting for 38.1% of the cases in the clamshell group and 45.2% of the cases in the VATS group. The overall distribution of primary diagnoses did not differ significantly between groups (*P* = 0.587). The median waiting time on the transplant list and the proportions of patients requiring mechanical ventilation or ECMO bridging were also comparable.

**Table 1:** Baseline patient characteristics

	Clamshell (*n* = 105)	VATS (*n* = 31)	*P*-value
Recipient data			
Age (years, median (IQR))	60.00 (52.00–65.00)	62.00 (51.00–68.00)	0.212
Sex (male, %)	43 (41.0)	12 (38.7)	0.823
BMI (mean ± SD)	20.67 ± 4.05	20.79 ± 3.46	0.187
Height (mean ± SD)	160.89 ± 14.13	163.40 ± 10.30	0.321
Weight (mean ± SD)	54.48 ± 14.50	55.85 ± 11.49	0.578
Comorbidity (*n*, %)			
HTN	34 (32.4)	9 (29.0)	0.725
DM	20 (19.0)	8 (25.8)	0.413
CAD	12 (11.7)	1 (3.2)	0.165
CVA	4 (3.8)	0 (0.0)	0.574
Diagnosis (*n*, %)			0.587
IPF	40 (38.1)	14 (45.2)	
CTD-ILD	29 (27.6)	7 (22.6)	
Other ILD	6 (5.7)	1 (3.2)	
COPD	4 (3.8)	0 (0.0)	
GVHD	10 (9.5)	1 (3.2)	
Others	16 (15.2)	8 (25.8)	
Waiting time (days, median (IQR))	157.00 (42.00–460.00)	135.00 (66.00–325.00)	0.839
BTT ECMO (*n*, %)	39 (37.1)	6 (19.4)	0.064
Mechanical ventilator (*n*, %)	36 (34.3)	5 (16.1)	0.064
Pulmonary hypertension (ePASP > 50 mmHg) (*n*, %)	56 (53.3)	18 (58.1)	0.642
ePASP (mmHg, median (IQR))	50.00 (39.00–62.00)	54.50 (36.75–73.00)	0.441
Donor data			
Height	166.25 ± 9.82	166.55 ± 10.19	0.964
Weight	64.26 ± 14.04	68.73 ± 14.78	0.688

*BMI* body mass index, *BTT* bridge to transplant, *CAD* coronary artery disease, *COPD* chronic obstructive pulmonary disease, *CTD-ILD* connective tissue disease-associated interstitial lung disease, *CVA* cerebrovascular accident, *DM* diabetes mellitus, *ECMO* extracorporeal membrane oxygenation, *ePASP* estimated pulmonary artery systolic pressure, *GVHD* graft-versus-host disease, *HTN* hypertension, *ILD* interstitial lung disease, *IQR* interquartile range, *SD* standard deviation

The median estimated pulmonary artery systolic pressure (ePASP) was 50.0 mmHg (IQR 39.0–62.0) in the clamshell group and 54.5 mmHg (IQR 36.7–73.0) in the VATS group (*P* = 0.441). Pulmonary hypertension (defined as ePASP ≥ 50 mmHg) was observed in 56 patients (53.3%) in the clamshell group and 18 patients (58.1%) in the VATS group, with no significant difference between groups (*P* = 0.642).

### Operative outcomes

Intraoperative and postoperative outcomes are summarized in Table 2. Compared with the VATS group, the clamshell group had significantly longer operation time (416.90 ± 80.94 vs. 319.23 ± 39.64 min; *P* < 0.001), total warm ischemic time (121.34 ± 24.38 vs. 107.52 ± 9.73 min; *P* = 0.001), and time from last reperfusion to skin closure (120.83 ± 33.10 vs. 68.45 ± 13.44 min; *P* < 0.001). Estimated blood loss was substantially greater in the clamshell group than in the VATS group (2788.57 ± 3364.82 vs. 832.26 ± 882.53 mL; *P* < 0.001), accompanied by significantly higher intraoperative transfusion requirements, including red blood cells (7.5 vs. 2.7 units; *P* < 0.001), fresh frozen plasma (4.0 vs. 0.6 units; *P* < 0.001), and platelets (0.7 vs. 0.1 units; *P* < 0.001).

**Table 2:** Perioperative outcomes

	Clamshell (*n* = 105)	VATS (*n* = 31)	*P*-value
Operation time (min, mean ± SD)	416.90 ± 80.94	319.23 ± 39.64	< 0.001
Total warm ischemic time (min, mean ± SD)	121.34 ± 24.38	107.52 ± 9.73	0.001
Last reperfusion to skin closure (min, mean ± SD)	120.83 ± 33.10	68.45 ± 13.44	< 0.001
EBL (ml, mean ± SD)	2788.57 ± 3364.82	832.26 ± 882.53	< 0.001
Intraoperative transfusion (*n*, %)	98 (93.3)	23 (74.2)	0.015
RBC (unit, mean ± SD)	7.49 ± 6.17	2.74 ± 2.71	< 0.001
FFP (unit, mean ± SD)	3.96 ± 5.40	0.58 ± 1.31	< 0.001
PLT (unit, mean ± SD)	0.68 ± 1.10	0.13 ± 0.50	< 0.001
Bleeding reoperation (*n*, %)	13 (12.4)	2 (6.5)	0.519
Delayed wound closure (*n*, %)	3 (2.9)	0 (0.0)	0.588
Superficial wound problem (*n*, %)	1 (1.0)	1 (3.2)	0.396
Reoperation for sternal dehiscence (*n*, %)	2 (1.9)	0 (0.0)	> 0.99
Airway stenting (*n*, %)	11 (10.5)	0 (0.0)	0.069
Postoperative ICU stay (d, median (IQR))	10 (6–14)	8 (6–10)	0.011
Postoperative hospital stay (d, median (IQR))	53 (33.50–99.50)	53 (41.00–103.00)	0.478
30-day mortality (*n*, %)	6 (5.7)	0 (0.0)	0.336

*EBL* estimated blood loss, *FFP* fresh frozen plasma, *ICU* intensive care unit, *RBC* red blood cell, *SD* standard deviation

Sternal dehiscence with suspected mediastinitis requiring surgical intervention occurred in two patients (1.9%) in the clamshell group only. Bronchial stenting was required in 11 patients (10.5%) in the clamshell group—10 due to stenosis and one due to anastomotic dehiscence. No patient in the VATS group required bronchial stenting; however, this difference was not statistically significant (*P* = 0.069). One patient in the VATS group developed an iatrogenic superficial femoral artery-common femoral vein arteriovenous fistula related to femoral cannulation, which was successfully managed with endovascular treatment. No cases of limb ischemia or major vascular injury were observe. The clamshell group had a longer postoperative intensive care unit (ICU) stay (median, 10 vs. 8 days; *P* = 0.011) than the VATS group. However, there were no significant differences in postoperative hospital length of stay or 30-day mortality between groups.

In multivariable analyses with robust standard errors (Table 3), the VATS approach remained independently associated with a significantly shorter operative time (*β* =  − 97.6 min; 95% CI − 121.1 to − 74.0; *P* < 0.001), reduced intraoperative blood loss (ratio 0.37; 95% CI 0.25 − 0.55; *P* < 0.001), and a shorter ICU length of stay (ratio 0.77; 95% CI 0.63–0.95; *P* = 0.016). These findings were consistent with the unadjusted analyses.

**Table 3:** Multivariable analysis of perioperative outcomes associated with the VATS approach

Outcome	Effect measure	Adjusted estimate (95% CI)	*P*-value
Operative time (min)	*β* (min)	− 97.6 ( − 121.1 to − 74.0)	< 0.001
ICU LOS (days)	Ratio	0.77 (0.63–0.95)	0.016
Estimated blood loss (mL)	Ratio	0.37 (0.25–0.55)	< 0.001

Multivariable linear and log-linear regression models with robust standard errors were used. Models were adjusted a priori for recipient age, sex, body mass index, pulmonary hypertension (ePASP ≥ 50 mmHg), preoperative mechanical ventilation, preoperative ECMO support, and primary diagnosis (IPF vs no-IPF)

*CI* confidence interval, *ECMO* extracorporeal membrane oxygenation, *ePASP* estimated pulmonary artery systolic pressure, *ICU* intensive care unit, *IPF* idiopathic pulmonary fibrosis, *LOS* length of stay

### Pulmonary function test

**Fig. 3:**
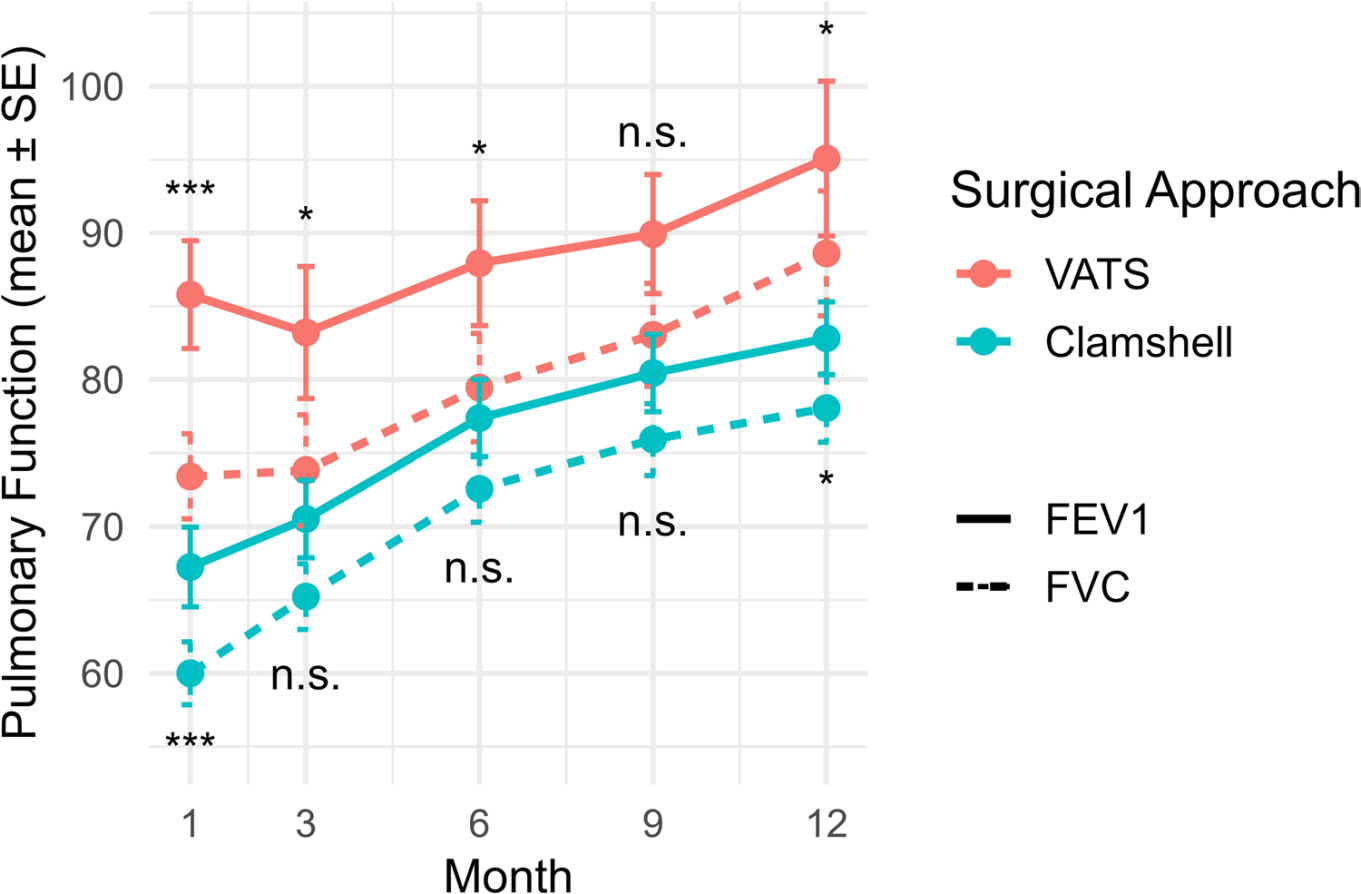
Trends in postoperative pulmonary function following lung transplantation by surgical approach. *, *P* < 0.05; **, *P* < 0.01; ***, *P* < 0.001; n.s. = not significant. *P*-values for FEV_1_ comparisons are displayed above the VATS curves, while *P*-values for FVC comparisons are shown below the Clamshell curves. *FEV*_1_ forced expiratory volume in 1 s, *FVC* forced vital capacity, *SE* standard error

Postoperative pulmonary function, assessed by FEV_1_ and FVC, was consistently better in the VATS group across all time points. Figure 3 illustrates the mean FEV_1_ and FVC values (± standard error) at 1, 3, 6, 9, and 12 months postoperatively for both groups. Patients in the VATS group exhibited significantly higher FEV_1_ and FVC at 1 month postoperatively (FEV_1_: 67.25 ± 22.55 vs. 85.79 ± 18.00; *P* < 0.001, FVC: 60.00 ± 17.81 vs. 73.42 ± 14.14; *P* < 0.001). These differences gradually attenuated over time but remained statistically significant at 12 months (FEV_1_: *P* = 0.05; FVC: *P* = 0.04).

Linear mixed-effects modeling was performed to evaluate longitudinal changes in pulmonary function over 12 months after transplantation (Table 4). At baseline (1 month postoperatively), the patients in the VATS group demonstrated significantly higher FEV_1_ and FVC values than did those in the clamshell group (FEV_1_ estimate = 15.13, *P* = 0.002; FVC estimate = 9.97, *P* = 0.020). Both FEV_1_ and FVC increased significantly over time in the clamshell group (slopes: 1.68 and 1.86% predicted per month, respectively), and while the VATS group showed numerically slower slopes (FEV_1_: 1.05%, FVC: 1.59%), only the interaction effect for FEV_1_ reached statistical significance (*P* = 0.022). These findings suggest that the clamshell group experienced a steeper recovery in FEV_1_, whereas longitudinal FVC improvement patterns were similar between groups (*P* = 0.223).

**Table 4:** Fixed effects estimates from linear mixed-effects model for postoperative pulmonary function (Reference group = Clamshell group, reference time = postoperative 1 month)

	Estimate	Std.Error	*t* value	*P*-value
FEV1				
(Intercept)	65.62	2.44	26.84	< 0.001
Group	15.13	4.89	3.10	0.002
Time	1.68	0.13	13.38	< 0.001
Group x time	− 0.63	0.28	− 2.30	0.022
FVC				
(Intercept)	59.54	2.12	28.07	< 0.001
Group	9.97	4.24	2.35	0.020
Time	1.86	0.10	18.29	< 0.001
Group x time	− 0.27	0.22	− 1.22	0.223

*FEV1* forced expiratory volume in 1 s, *FVC* forced vital capacity

## Discussion

This study demonstrated that bilateral lung transplantation via the VATS approach was associated with shorter operative time, reduced intraoperative blood loss, and lower transfusion requirements compared with the conventional clamshell incision. Importantly, the difference in operative duration appeared to be largely driven by the post-reperfusion phase, including hemostasis and chest wall closure, rather than by differences in implantation efficiency, as reflected by comparable warm ischemic times. These findings suggest that incision-related factors may account for a substantial proportion of total operative time, without evidence of increased ischemic exposure or compromised early safety outcomes.

Furthermore, patients in the VATS group demonstrated improved postoperative pulmonary function compared with those in the clamshell group. Although causal inference is limited by the retrospective design, preservation of chest wall integrity and reduced surgical trauma may have contributed to more favorable early functional recovery.

Avoiding sternal division is a key advantage of the VATS approach. Preservation of chest wall integrity resulted in fewer sternal wound complications; in the clamshell group, 1.9% of patients experienced sternal dehiscence requiring reoperation, whereas no such events occurred in the VATS group. A stable chest wall may also facilitate earlier rehabilitation and safer patient positioning when required. Although bilateral thoracotomy has been proposed as a sternal-sparing alternative, previous reports typically described large incisions and relied on open instrumentation. [4] In contrast, our technique integrates thoracoscopic visualization, endoscopic vascular clamps, and VATS instruments to perform critical hilar dissection and anastomosis through mini-thoracotomy incisions. This approach preserves the benefits of minimally invasive surgery while ensuring adequate exposure for safe transplantation.

Recently, robotic approaches to lung transplantation have emerged as minimally invasive alternatives, demonstrating potential benefits such as reduced postoperative pain, shorter ICU stays, and improved pulmonary function. [8] Comparable advantages were observed in our cohort using the VATS approach. Compared with robot-assisted lung transplantation, VATS offers practical advantages; it is better suited to the smaller chest cavities common in Asian patients and facilitates the handling of marginal donor lungs with fewer access ports and without the need for robotic systems. The reduced number of ports and direct access via mini-thoracotomies simplify the procedure in patients with anatomical constraints. Moreover, VATS does not require expensive robotic platforms, potentially enhancing its accessibility. However, VATS has certain limitations. It requires an experienced thoracoscopic assistant (scopist) and sufficient intrathoracic working space for safe camera maneuverability and instrument handling. Specialized equipment, including endoscopic vascular clamps for pulmonary artery and left atrium control, as well as custom lung insertion bags, is essential for facilitating allograft implantation through a limited working window. Unlike robotic approaches, VATS relies on conventional thoracic instruments and techniques, making it more readily adoptable and reducing the need for specialized training or costly infrastructure. Given that VATS is already a standard thoracic surgical technique, this approach may be more feasible and scalable across transplant centers.

Notably, our cohort represented a critically ill population, with a higher prevalence of pulmonary hypertension and greater reliance on preoperative ECMO support compared with previous minimally invasive lung transplantation cohorts. [9] Whereas earlier reports described intraoperative cardiopulmonary support in approximately 50% of cases, all patients in our study underwent transplantation under ECMO support. This reflects a high prevalence of IPF with pulmonary hypertension and frequent need for ECMO bridging. Despite this elevated baseline risk, VATS was associated with favorable intraoperative and early postoperative outcomes, supporting its feasibility in high-risk patients.

Peripheral ECMO has been regarded as a potential limitation of minimally invasive lung transplantation because of concerns regarding vascular access-related complications and differential hypoxemia. Large contemporary series have demonstrated higher rates of vascular complications—particularly acute limb ischemia—with peripheral venoarterial ECMO, highlighting the importance of meticulous cannulation and limb perfusion management. [10] Expert consensus statements similarly acknowledge a preference for central ECMO in many settings, while emphasizing that the choice of cannulation strategy should be individualized based on institutional expertise and patient-specific factors. [11]

In present study, peripheral ECMO was applied using a protocol-driven strategy incorporating optimized venous cannulation positioning, phase-specific ECMO management during sequential implantation, selective use of distal perfusion catheters, and continuous lower-limb monitoring with near-infrared spectroscopy. Prior studies have shown that near-infrared spectroscopy facilitates early detection of limb ischemia and reduces severe limb complications during peripheral VA ECMO support. [12] With these measures, peripheral ECMO was safely implemented, with no cases of limb ischemia or clinically significant differential hypoxemia; one patient in the VAT group developed an iatrogenic superficial femoral artery-common femoral vein arteriovenous fistula, which was successfully treated with endovascular intervention. These findings support the feasibility of peripheral ECMO in minimally invasive lung transplantation when comprehensive management protocols are employed.

Minimally invasive approaches are often considered unsuitable for patients with prior thoracic surgery, pleurodesis, or severe pleural adhesions. However, in our experience, these factors do not contraindicate VATS. Thoracoscopic visualization enables safer and more controlled adhesiolysis, particularly in regions that are difficult to access through open techniques, such as the apex, posterior mediastinum, and diaphragm. This observation parallels developments in thoracic oncology, where VATS and robotic techniques are increasingly adopted for complex reoperative cases. [7]

Preservation of thoracic stability has been associated with improve pulmonary function, reduced postoperative pain, and enhanced mobility. [9, 13] Our findings are consistent with these observations and highlight the potential importance of minimizing surgical trauma, particularly in patients with restrictive chest walls.

Despite its retrospective design, this study benefits from a consistent institutional protocol with routine ECMO support, detailed intraoperative time metrics allowing phase-specific analysis, and a standardized surgical team with a single high-volume center, minimizing inter-institutional variability.

This study is limited by its retrospective, single-center design and the relatively small size of the VATS cohort. In addition, the VATS approach was introduced later during the study period (April 2024), reflecting a time-based institutional transition rather than randomized allocation. Although VATS was adopted as the default approach after its introduction and was not restricted to fitter recipients, residual selection bias and temporal effects cannot be excluded. Standardized postoperative pain assessments, such as numeric rating scale or visual analog scale scores at predefined time points, as well as comprehensive opioid-equivalent consumption data, were not consistently available in our institutional database, precluding a robust comparative analysis of postoperative pain—an important endpoint of minimally invasive surgery. Nevertheless, the consistent intraoperative advantages and absence of major complications underscore the potential clinical value of the VATS approach. Further multicenter studies with larger cohorts and long-term follow-up are warranted to validate these findings and refine patient selection criteria.

In conclusion, bilateral lung transplantation using the VATS approach appears to be a safe and effective alternative to the traditional clamshell incision, offering reduced surgical trauma and favorable early postoperative outcomes. This technique may broaden the options for less invasive lung transplantation, particularly in centers with expertise in advanced thoracoscopic procedures.

## Supplementary Information

Below is the link to the electronic supplementary material.

Supplementary file1 (MP4 76883 KB)
